# GNPNAT1 is a potential biomarker correlated with immune infiltration and immunotherapy outcome in breast cancer

**DOI:** 10.3389/fimmu.2023.1152678

**Published:** 2023-05-05

**Authors:** Renjie Yuan, Yulu Zhang, Yange Wang, Hongling Chen, Ruiming Zhang, Zhiyuan Hu, Chengsen Chai, Tingmei Chen

**Affiliations:** ^1^ Key Laboratory of Clinical Laboratory Diagnostics (Ministry of Education), College of Laboratory Medicine, Chongqing Medical University, Chongqing, China; ^2^ Chinese Academy of Sciences (CAS) Key Laboratory of Standardization and Measurement for Nanotechnology, Chinese Academy of Sciences (CAS) Key Laboratory for Biomedical Effects of Nanomaterials and Nanosafety, Chinese Academy of Sciences (CAS) Center for Excellence in Nanoscience, National Center for Nanoscience and Technology, Beijing, China

**Keywords:** GNPNAT1, breast cancer, biomarker, tumor-infiltration immune cells, immune checkpoints inhibitors, immunotherapy response

## Abstract

**Background:**

Glucosamine 6-phosphate N-acetyltransferase (GNPNAT1) is a crucial enzyme involving hexosamine biosynthesis pathway and is upregulated in breast cancer (BRCA). However, its biological function and mechanism on patients in BRCA have not been investigated.

**Methods:**

In this study, the differential expression of GNPNAT1 was analyzed between BRCA tissues and normal breast tissues using the Cancer Genome Atlas (TCGA) database and Gene Expression Omnibus (GEO) database, which was validated by quantitative real-time polymerase chain reaction, Western blot and immunohistochemistry. Then, the potential clinical value of GNPNAT1 in BRCA was investigated based on TCGA database. Functional enrichment analyses, including Gene Ontology, Kyoto Encyclopedia of Genes and Genomes, Gene Set Variation Analysis, were performed to explore the potential signaling pathways and biological functions involved in GNPNAT1 in BRCA. Tumor immune infiltration was analyzed using ESTIMATE, CIBERSORT and TISIDB database; and immune therapy response scores were assessed using TIDE. Finally, Western blot, Cell counting kit-8 and Transwell assay were used to determine the proliferation and invasion abilities of breast cancer cells with GNPNAT1 knockdown.

**Results:**

GNPNAT1 was up-regulated in BRCA tissues compared with normal tissues which was subsequently verified in different cell lines and clinical tissue samples. Based on TCGA and GEO, the overexpression of GNPNAT1 in BRCA contributed to a significant decline in overall survive and disease specific survive. Functional enrichment analyses indicated that the enriched pathways in high GNPNAT1 expression group included citrate cycle, N-glycan biosynthesis, DNA repair, and basal transcription factors. Moreover, the overexpression of GNPNAT1 was negatively correlated with immunotherapy response and the levels of immune cell infiltration of CD8+ T cells, B cells, natural killer cells, dendritic cells and macrophages. Knockdown of GNPNAT1 impairs the proliferation and invasion abilities of breast cancer cells.

**Conclusion:**

GNPNAT1 is a potential diagnostic, prognostic biomarker and novel target for intervention in BRCA.

## 1 Introduction

Breast cancer (BRCA) has surpassed lung cancer as the most commonly diagnosed malignancy worldwide, and it is the leading cause of cancer-related death among women (1). Breast cancer is a complex disease that displays a large degree of heterogeneity (2, 3). Due to the immunohistochemical expression of the following receptors: estrogen receptor (ER), progesterone receptor (PR) and human epidermal growth factor receptor 2 (HER2), this heterogeneous disease is categorized into three groups: hormone receptors (HR) positive, HER2 positive, and triple-negative (4). HR positive tumors are characterized by the presence of HR and the absence of HER2, hence these patients present ideal efficacy to hormone therapy (tamoxifen or aromarase inhibitors). HER2 positive breast cancer is an aggressive subtype, its standard first-line treatment consists of HER2 targeted therapy, including trastuzumab, and pertuzumab. Triple Negative Breast Cancers (TNBC), without the expression of HR, are a group with only chemotherapy and immunotherapy options (5). Despite significant improvements in the diagnosis and treatment of breast cancer, there are still a large number of patients who are resistant to therapy or relapse after treatment. Therefore, identifying a universal clinical biomarker and therapeutic target will benefit patients in BRCA.

Glucosamine 6-phosphate N-acetyltransferase (GNPNAT1), also called GNA1, encodes a crucial enzyme catalyzing the formation of N-acetylglucosamine-6-phosphate from acetyl-CoA and the acceptor substrate glucosamine-6-phosphate (6, 7), which is an essential intermediate in hexosamine biosynthesis pathway (HBP). The end product of HBP is UDP-N-acetylglucosamine, and its expression level is known to affect protein O−GlcNAcylation by O-GlcNAc transferase. Previous studies found that GNPNAT1 is a potential biomarker to predict sensitivity to radiotherapy of ER positive BRCA patients (8). Furthermore, researchers proved that GNPNAT1 is a promising diagnostic and prognostic biomarker and correlates with immune infiltration in lung adenocarcinoma (9–11). However, its clinical value and potential mechanism in BRCA are still unclear.

In this study, we first analyzed the differential expression of GNPNAT1 between BRCA tissues and normal breast tissues and elucidated its clinical implications based on the Cancer Genome Atlas (TCGA) and Gene Expression Omnibus (GEO) database, which was also confirmed by quantitative real-time polymerase chain reaction (qRT-PCR), Western blot and immunohistochemistry (IHC). Furthermore, we investigated the correlation between GNPNAT1 expression and its potential involved signaling pathways, immune infiltration levels and immunotherapy response. Additionally, Knockdown of GNPNAT1 impairs the proliferation and invasion abilities of breast cancer cells. Based on these results, we could better understand the potential role of GNPNAT1 in BRCA, and our study provided a new perspective of GNPNAT1 for the potential diagnosis, prognosis and even treatment of patients in BRCA in the future.

## 2 Materials and methods

### 2.1 Data acquisition

The expression profiles and the corresponding clinical information of BRCA patients were downloaded from TCGA via GDC portal (https://portal.gdc.cancer.gov/). Two BRCA datasets (GSE10810 and GSE70591) were downloaded from the GEO database (https://www.ncbi.nlm.nih.gov/geo/). Then, we used TIMER 2.0 (http://timer.cistrome.org/) to analyze pan-cancer expression pattern of GNPNAT1, and we used UCALAN (http://ualcan.path.uab.edu/) and HPA (https://www.proteinatlas.org/) to compare protein expression of GNPNAT1 between BRCA tissues and normal tissues.

### 2.2 Receiver operating characteristic curves and survival analysis

ROC curves were used to evaluate the diagnostic efficiency of GNPNAT1. We generated ROC curves using the “pROC” R package (12). Area under curve (AUC) was used as the following criteria: 0.50-0.60 = fail, 0.60-0.70 = poor, 0.70-0.80 = fair, 0.80-0.90 = good and 0.90-1 = excellent to evaluate diagnostic performance. Then, we defined patients with the expression level of GNPNAT1 higher than median as the high_GNPNAT1 group and the remainder as the low_GNPNAT1 group. Survival statistics were plotted by GEPIA 2 database (https://gepia2.cancer-pku.cn/), K-M plotter database (https://kmplot.com/) and analyzed using the Log-Rank test. Cox regression model was applied for regression analysis.

### 2.3 Identification of differentially expressed genes and functional enrichment analysis

Firstly, the 1083 BRCA patients in TCGA were divided into two groups by the median expression level of GNPNAT1. DEGs were identified using “DESeq2” R package (13). False positive results were corrected using the adjusted P value. The screening thresholds for differentially expressed genes (DEGs) were defined as follows: adjusted P value < 0.05 and |log2 (fold change) | > 1. Gene Ontology (GO) and Kyoto encyclopedia of genes and genomes (KEGG) analyses were conducted using the “clusterProfiler” R package (14). Adjusted P value < 0.05 is considered statistically significant in the enrichment results. Then, we applied Gene Set Variation Analysis (GSVA) to calculate enrichment scores in each patient for the 186 KEGG pathways described in the molecular signature database (MSigDB v7.5.1), as implemented in the “GSVA” R package (15). GSVA output was then submitted to “limma” to determine differentially expressed pathways (16).

### 2.4 Tumor immune infiltration analysis

We evaluated the levels of the immune cell infiltration, the stromal content, the stromal-immune comprehensive score and tumor purity for each BRCA sample by “ESTIMATE” R package (17). Then, a total of 22 immune cells were used to calculate the level of immune infiltration, and the relative enrichment score of these immune cells in breast cancer was assessed by “CIBERSORT” R package (18). We used TISIDB database (http://cis.hku.hk/TISIDB/) to determine the Spearman correlations between GNPNAT1 expression and Tumor-infiltrating Immune cells (TILs), major histocompatibility complex (MHC) molecule abundance in BRCA.

### 2.5 Immunotherapy response analysis

Use the TIDE algorithm to calculate immunotherapy response score that constructed by Jiang et al. (19), which based on the mRNA expression signature. TIDE uses a set of markers to evaluate two different mechanisms of tumor immune escape, including the dysfunction of tumor infiltrating cytotoxic T lymphocytes (CTL) and the rejection of CTL by immunosuppressive factors. The high TIDE score means that immune checkpoint blocking therapy has poor efficacy.

### 2.6 Cell lines and cells culture

Human non-transformed mammary epithelial cell line MCF-10A and breast cancer cell lines, including MCF-7, ZR-75-1, SK-BR-3, MDA-MB-231, BT549, Hs578t, in this study were purchased from National Collection of Authenticated Cell Cultures (China). All cell lines were cultured under 37°C, 5% CO2 condition.

### 2.7 RNA extraction and qRT-PCR

RNA was extracted from whole cell lysates with Trizol reagent (Takara, Japan) and was reverse-transcribed with the PrimeScript™ RT reagent Kit (Takara, Japan) according to the instructions of manufacturer. All qRT-PCR was performed with the SYBR Premix Ex Taq II (TaKaRa, Japan). The primers were as follows: forward primer 5’- CACCATTGGCAATGAGCGGTTC-3’ and reverse primer 5’- AGGTCTTTGCGGATGTCCACGT-3’ for Actin Beta; forward primer 5’- ATTTGGGTCGCAGTTCTTG-3’ and reverse primer 5’- TGCCTTGACATTCTCGATGGT-3’ for Ubiquitin C; forward primer 5’-AGGGCCTCTACGGTTCCTGT-3’ and reverse primer 5’-GTGTTGGGGAAATGGCTGGA-3’ for GNPNAT1. Reference genes used in each qPCR run were Actin Beta and Ubiquitin C (20).

### 2.8 Western blot

Total protein was extracted by RIPA buffer (Beyotime, China) containing 1% PMSF (Beyotime, China) and was quantified using a BCA Kit (Beyotime, China) according to the instructions of the manufacturer. 40 mg of protein was fractionated by SDS-PAGE, transferred onto 0.45 μm PVDF membrane (Millipore, USA). The membranes were blocked with defatted milk powder (Biosharp, China) at room temperature for one hour and incubated with the appropriate primary antibodies. The specific primary antibodies against GNPNAT1 (16282-1-AP, Proteintech, 1:1000), α-Tubulin (66031-1-Ig, Proteintech, 1:20000), c-Myc (67447-1-Ig, Proteintech, 1:5000), Cyclin D1 (WL01435a, Wanlei, 1:1000), Bcl-2 (BF9103, Affinity, 1:1000), Bax (2772, CST, 1:1000), E-cadherin (ET1607-75, Huabio, 1:1000), N-cadherin (ET1607-37, Huabio, 1:1000), Vimentin (BS1491, Bioworlde, 1:1000) were used and incubated at 4°C overnight. After being washed for three times in TBST, the membrane was incubated with corresponding HRP-conjugated secondary antibodies (BL001A and BL003A, Biosharp, 1:10000) at room temperature for one hour. Immunoblots were visualized by gel and blot imaging system (Bio-Rad, USA) with enhanced chemiluminescence assay (Millipore, USA).

### 2.9 Pathological sample collection and immunohistochemistry

A total of 16 pairs of paraffin-embedded breast cancer tissues and their matched para-tumor tissues were collected at the Pathology Department of the First Affiliated Hospital of Chongqing Medical University. These samples include 5 pairs of HR positive patients, 6 pairs of HER2 positive patients and 5 pairs of TNBC patients. Breast cancer tissues and para-tumor tissues were fixed in 10% neutralized formaldehyde for 24 hours and then embedded in paraffin. The paraffin-embedded samples were cut into 4-μm-thick sections. The primary antibody against GNPNAT1 (16282-1-AP, Proteintech; 1:200) was incubated overnight at 4°C. Afterwards, the slides were incubated with secondary antibody at room temperature for one hour and stained with DAB substrate, followed by hematoxylin counterstaining. IHC optical density scores were calculated to quantify protein expression in ImageJ (21).

### 2.10 Cell transfection

To generate MCF-7 and MDA-MB-231 cells with GNPNAT1 knockdown, cells transfection was performed by Lipofectamine 2000 (Invitrogen, USA) according to the manufacturer’s instructions. The siRNA targeting GNPNAT1 and control siRNA were purchased from Tsingke Biotechnology Co., Ltd. (China). The sequences for siGNPNAT1 are 5′-GAGUCAGAAUACAGCUACA(dT)(dT)-3′.

### 2.11 Cell counting kit-8 assay

The CCK-8 (Biosharp, China) assay was used to detect the cell proliferation ability. MCF-7 and MDA-MB-231 were seeded in 96-well plates at a density of 5×10^3^ cells per well and 2×10^3^ cells per well, respectively. The 10% CCK-8 regent was added after incubating the cells for blank, 0, 24, 48 and 72h, measuring the optical density (OD) value at 450nm and 650nm one hour later. The OD values used for analysis have subtracted the average OD value of blank wells.

### 2.12 Transwell assay

The MCF-7 and MDA-MB-231 cells with good morphology and growth were selected, digested with trypsin, and resuspended in serum free medium for later use. Matrigel (1: 8 dilutions; Corning, USA), and 200μL of cell suspension containing 2×10^4^ (MDA-MB-231) or 5×10^4^ (MCF-7) cells were added to the upper layer of the Transwell chamber, and 600μL culture medium with 20% serum was added to the lower chamber. After culturing for 24h (MDA-MB-231) or 72h (MCF-7), invading cells were fixed, stained with crystal violet, imaged, and counted by ImageJ (threshold range of cells counting: 180-infinity pixels^2).

### 2.13 Bioinformatics analysis and statistical analysis

**Figure 1:**
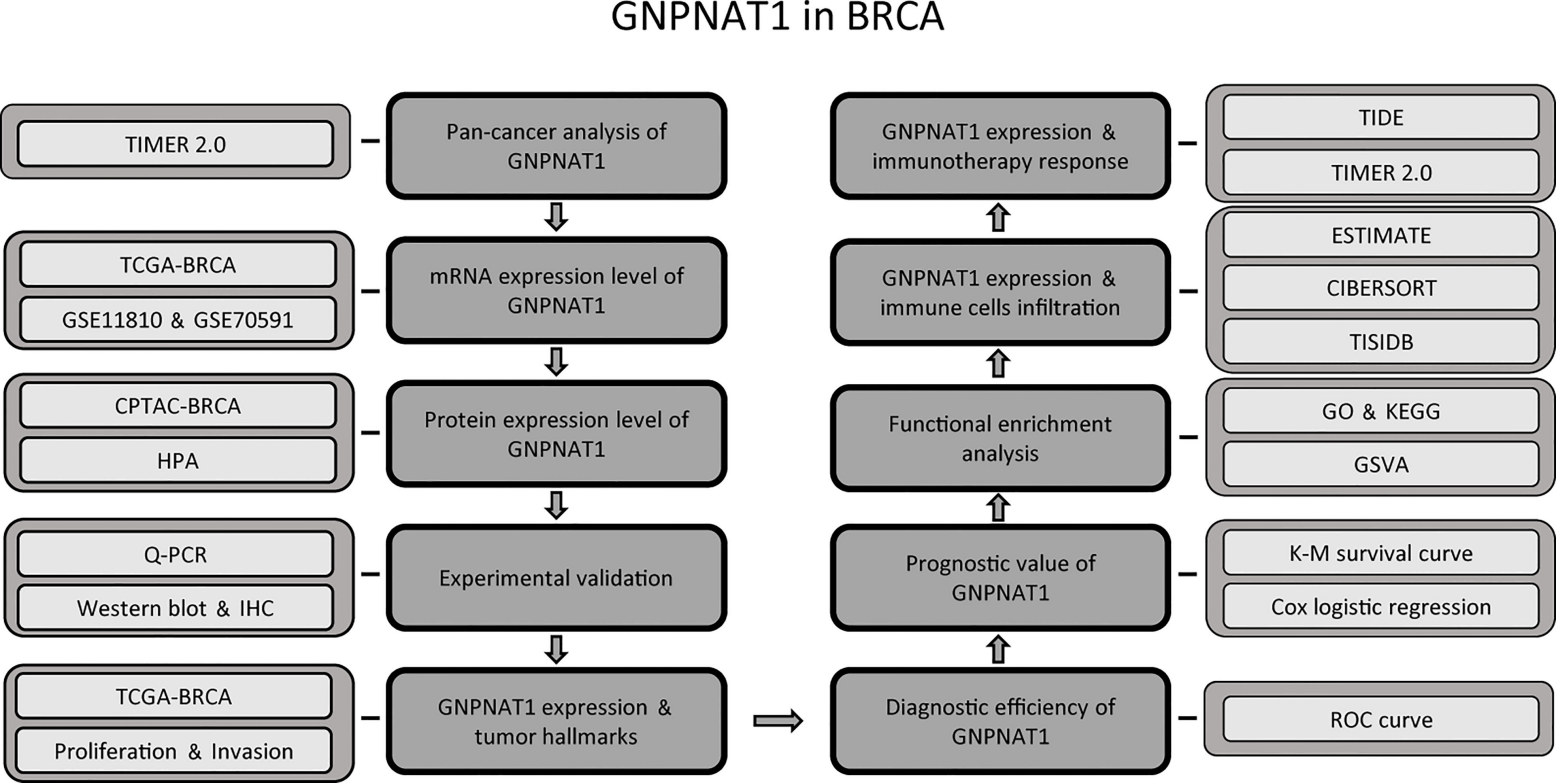
The flowchart of this study.

All analyses were done in R (v4.0.2) or GraphPad Prism (v9.4.0). The results of wet experiments were shown as mean ± standard deviation, n = 3. The methods of computing statistical significance were presented in corresponding figure legends and p-value was annotated by the number of stars (*: p-value < 0.05; **: p-value <0.01; ***: p-value <0.001; ****: p-value <0.0001). The flow diagram of this study was presented in **Figure 1**.

## 3 Results

### 3.1 The expression of GNPNAT1 in pan−cancers and BRCA

**Figure 2:**
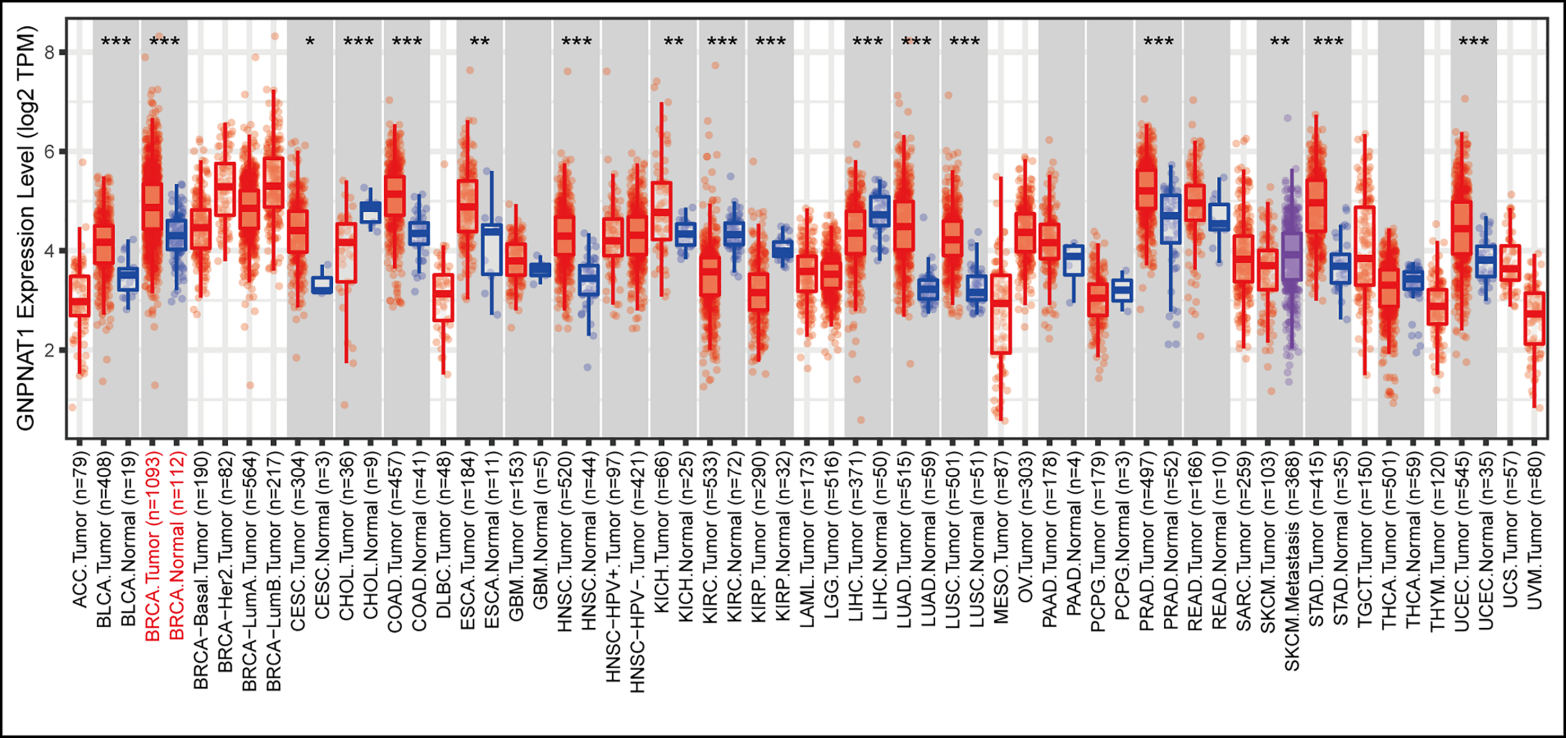
The expression pattern of GNPNAT1 in types of cancer via TIMER 2.0 database. The statistical significance computed by Wilcoxon rank sum test is annotated by the number of stars (*p-value < 0.05; **p-value <0.01; ***p-value <0.001).

Initially, Comparing GNPNAT1 expression between normal tissues and tumor samples from TCGA database, we found that GNPNAT1 was significantly up-regulated in many types of cancer, including BRCA (**Figure 2**).

**Figure 3:**
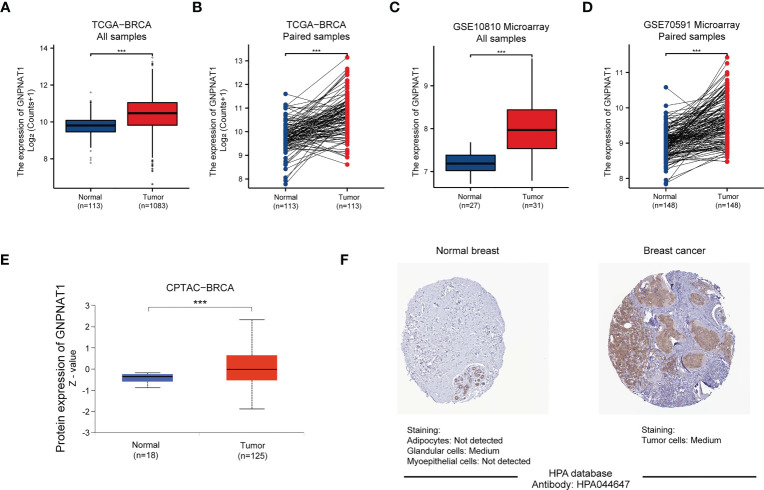
The mRNA and protein expression level of GNPNAT1 in BRCA patients. **(A)** The mRNA expression of GNPNAT1 significantly upregulated in BRCA compared with normal tissues from TCGA-BRCA (Wilcoxon rank sum test). **(B)** The mRNA expression of GNPNAT1 between normal tissues and matched tumor tissues from TCGA-BRCA (Paired samples t-test). **(C, D)** The mRNA expression of GNPNAT1 significantly upregulated in BRCA compared with normal tissues in two GEO cohorts (Wilcoxon rank sum test and Paired samples t-test). **(E)** The protein expression of GNPNAT1 significantly upregulated in BRCA compared with normal tissues in CPTAC cohort (Wilcoxon rank sum test). **(F)** The protein expression of GNPNAT1 in normal breast and BRCA tissue were visualized by immunohistochemical staining from HPA database. (***p-value <0.001).

Then, we analyzed the mRNA expression level of GNPNAT1 via TCGA cohort. The significant up-regulation of GNPNAT1 expression was found in breast cancer tissues compared with normal tissues (**Figure 3A**). In addition, GNPNAT1 was also highly expressed in 113 paired breast cancer tissues (**Figure 3B**). To further confirm the mRNA expression of GNPNAT1, the similar results was also found in two GEO cohorts (**Figures 3C, D**).

We also used UALCAN and HPA online database to identify protein levels of GNPNAT1 in BRCA. Based on CPTAC database, protein expression level of GNPNAT1 was elevated in BRCA tissues compared with normal tissues (**Figure 3E**). A higher level of expression of GNPNAT1 protein was also found in the BRCA tissues than that in the normal tissues, as visualized by the representative IHC images from the HPA database (**Figure 3F**).

In conclusion, the mRNA and protein expression level of GNPNAT1 were significantly elevated in BRCA tissues compared with normal breast tissues.

### 3.2 Validation of GNPNAT1 expression in cell lines and clinical samples

**Figure 4:**
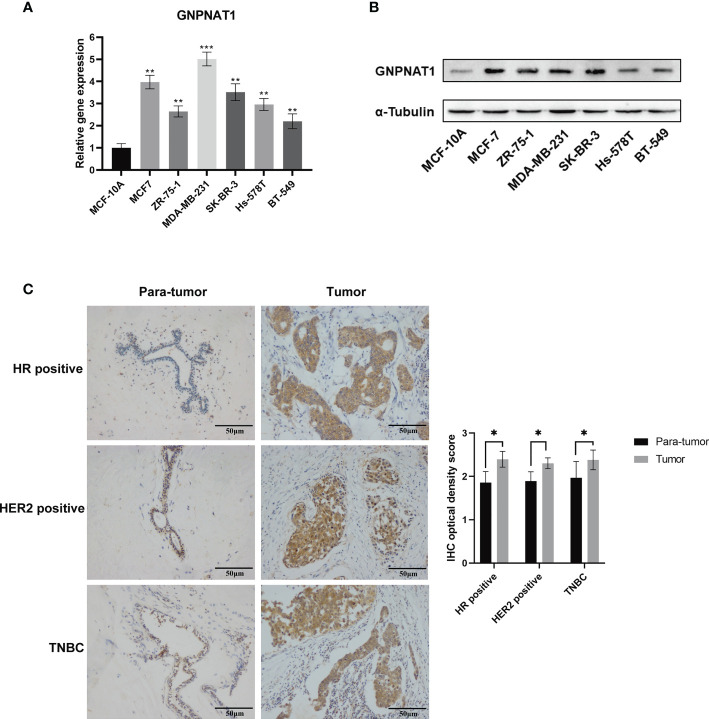
Validation of GNPNAT1 expression in cell lines and clinical tissues samples. **(A)** qRT-PCR (Independent samples t-test) and **(B)** Western blot was conducted in different breast cancer cell lines. **(C)** Representative microscopic images (200x magnification, scale bar 50 μm) demonstrate high expression of GNPNAT1 in BRCA tissues confirmed by IHC; the result of quantification of GNPNAT1 expression in IHC images is shown in the right part (Paired samples t-test). (*p-value < 0.05; **p-value <0.01; ***p-value <0.001).

To further identify the expression of GNPNAT1, we conducted qRT-PCR and Western blot on different cell lines, including one non-transformed mammary epithelial cell line and six breast cancer cell lines. The results indicated that mRNA and protein expression level of GNPNAT1 was significantly elevated in breast cancer cell lines compared with mammary epithelial cell line (**Figures 4A, B**).

The IHC staining was carried out on a cohort comprising 16 cases of primary breast cancer tissues paired with para-tumor tissues, and the expression of GNPNAT1 was up-regulated in 87.5% (14/16) of breast cancer tissues via IHC optical density score. Representative images of different subtypes were presented in **Figure 4C** separately, and quantization of results were shown in the right part.

These results indicated that the expression level of GNPNAT1 was significantly up-regulated in every subtype of BRCA cell lines and clinical tissues, and up-regulation of GNPNAT1 was not a specific subtype dependent.

### 3.3 Relationship between the expression level of GNPNAT1 and hallmarks of BRCA

**Figure 5:**
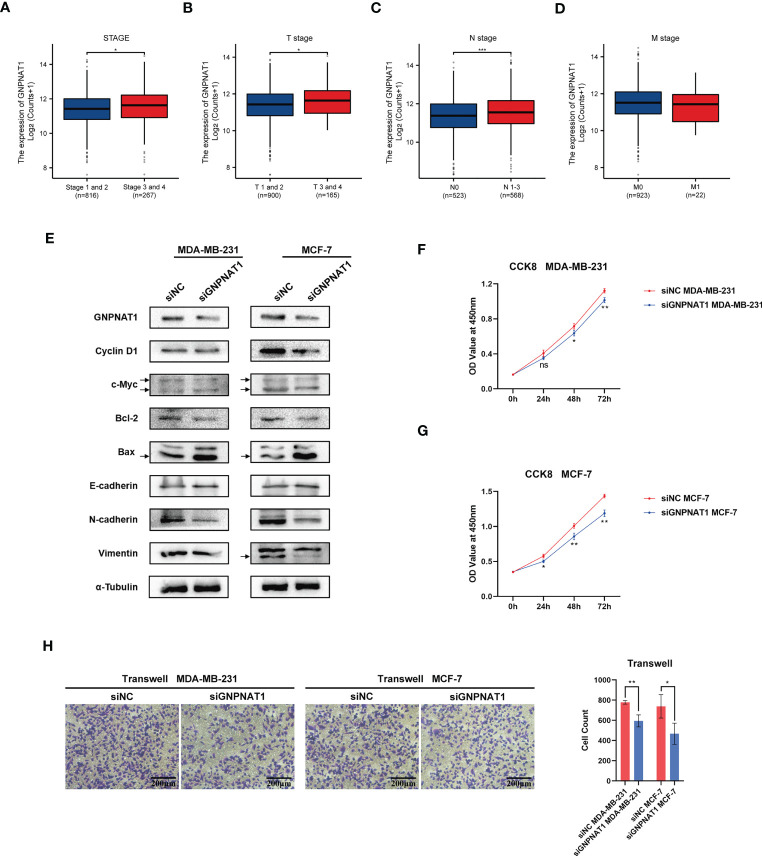
Association between the expression level of GNPNAT1 and hallmarks in BRCA. Association of GNPNAT1 mRNA expression and **(A)** stage, **(B)** T stage, **(C)** N stage, **(D)** M stage from TCGA database (Wilcoxon rank sum test). **(E)** Effect of GNPNAT1 knockdown on proliferation and invasion of MCF-7 and MDA-MB-231 were detected by Western blot analysis. Effect of GNPNAT1 knockdown on proliferation of **(F)** MDA‐MB‐231 and **(G)** MCF‐7 were detected by CCK‐8 assay (Independent samples t-test). **(H)** Effect of GNPNAT1 knockdown on invasion of MDA-MB-231 and MCF-7 were detected by Transwell assay (100x magnification, scale bar 200 μm), the result of cell counting is shown in the right part (Independent samples t-test). (*p-value < 0.05; **p-value <0.01; ***p-value <0.001).

To determine the relationship between the expression of the GNPNAT1 and hallmarks of BRCA, we firstly analyzed the clinical pathological characteristics of BRCA patients from TCGA. The mRNA expression level of the GNPNAT1 was significantly correlated with the stage, tumor stage and node stage, but it was nothing to do with metastasis stage (**Figure 5A-D**).

Due to the above results, we subsequently validated the relationship between the expression level of GNPNAT1 and proliferation and invasion abilities *in vitro*. We detected a serious of proteins related to cell proliferation and invasion. Knockdown of GNPNAT1 decreased the expression of c-Myc, CyclinD1 and Bcl-2, while Bax was upregulated; meanwhile, N-cadherin and Vimentin were downregulated but E-cadherin was upregulated as the knockdown of GNPNAT1 (**Figure 5E**). CCK-8 assay was used to detect the cell proliferation. As results indicated, knockdown of GNPNAT1 remarkably impaired the proliferation of MDA-MB-231 and MCF-7 (**Figures 5F, G**). Transwell assay was used to detect the cell invasion. As we anticipated, knockdown of GNPNAT1 attenuated the invasion of MDA-MB-231 and MCF-7 (**Figure 5H**).

To summarize, these results showed that GNPNAT1 might be associated with tumor progression and regional lymph node metastasis in BRCA patients, and knockdown of GNPNAT1 impairs the proliferation and invasion abilities of BRCA *in vitro*.

### 3.4 Potential diagnostic and prognostic value of GNPNAT1 in BRCA

Since GNPNAT1 was differentially expressed between breast cancer and normal tissues, we considered whether GNPNAT1 had potential diagnostic and prognostic value in BRCA.

**Figure 6:**
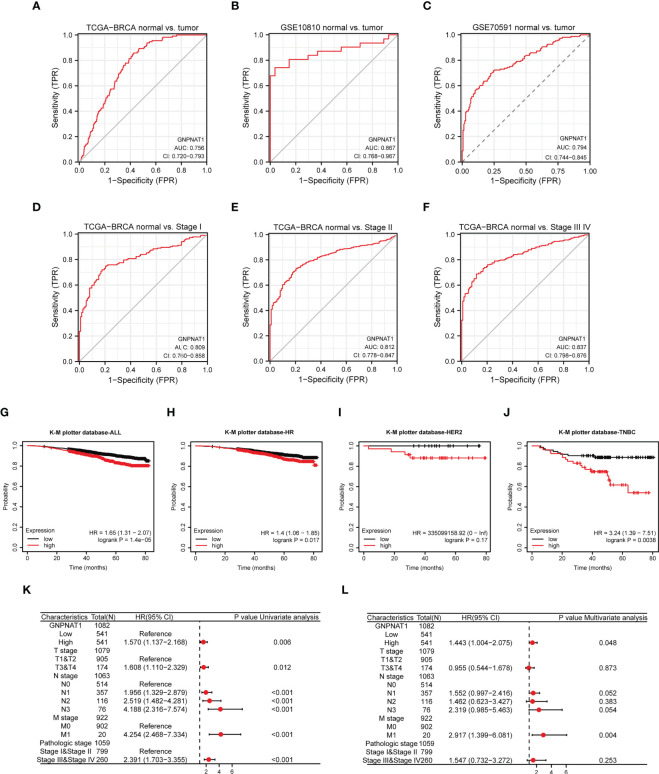
Potential clinical value of GNPNAT1 in BRCA patients. **(A–C)** ROC curves of GNPNAT1 expression in TCGA-BRCA and GEO cohorts. **(D–F)** ROC curves of GNPNAT1 expression in TCGA-BRCA cohort with different pathological stages. The Kaplan–Meier curves about the correlation between GNPNAT1 expression and overall survive of **(G)** all patients, **(H)** HR positive, **(I) **HER2 positive, and **(J)** TNBC of in TCGA database (Log-rank test) via K-M plotter. **(K, L)** Univariate and multivariate Cox analyses of GNPNAT1 and pathological characteristics.

Firstly, we used ROC curves to verify the diagnostic value of GNPNAT1. The results indicated that the area under the curve (AUC) of GNPNAT1 was 0.756, 0.867, 0.794 for TCGA, GSE10810 and GSE70591 cohort respectively (**Figures 6A–C**). Also, we examined the expression of GNPNAT1 at various stages in TCGA cohort with AUC values of 0.809, 0.812 and 0.837 for stages I, II, III and IV respectively (**Figures 6D–F**).

Furthermore, we analyzed the relationship between GNPNAT1 expression and prognosis of BRCA patients via K-M plotter database. Compared with the low GNPNAT1 expression group, the overall survive of the high GNPNAT1 expression group exhibited a significantly worse prognosis (**Figure 6G**). In different subtypes of BRCA patients, HR positive and TNBC patients had a significantly worse prognosis, while HER2 patients were not (**Figures 6H–J**). In **Supplement Figures 1A–D**, we re-access survive curve of BRCA patients via GEPIA 2 database, and results indicate that HER2 patients also had a significantly worse prognosis.

In addition, we evaluated the prognostic effect of GNPNAT1 expression in BRCA patients by using Cox regression analysis; in multicox analysis, GNPNAT1 was found as an independent predictor for overall survival in BRCA patients (**Figures 6K, L**).

These results suggested that GNPNAT1 expression have potential to be a diagnostic and prognostic biomarker in BRCA patients.

### 3.5 Identification of DEGs in BRCA and functional inference analysis

**Figure 7:**
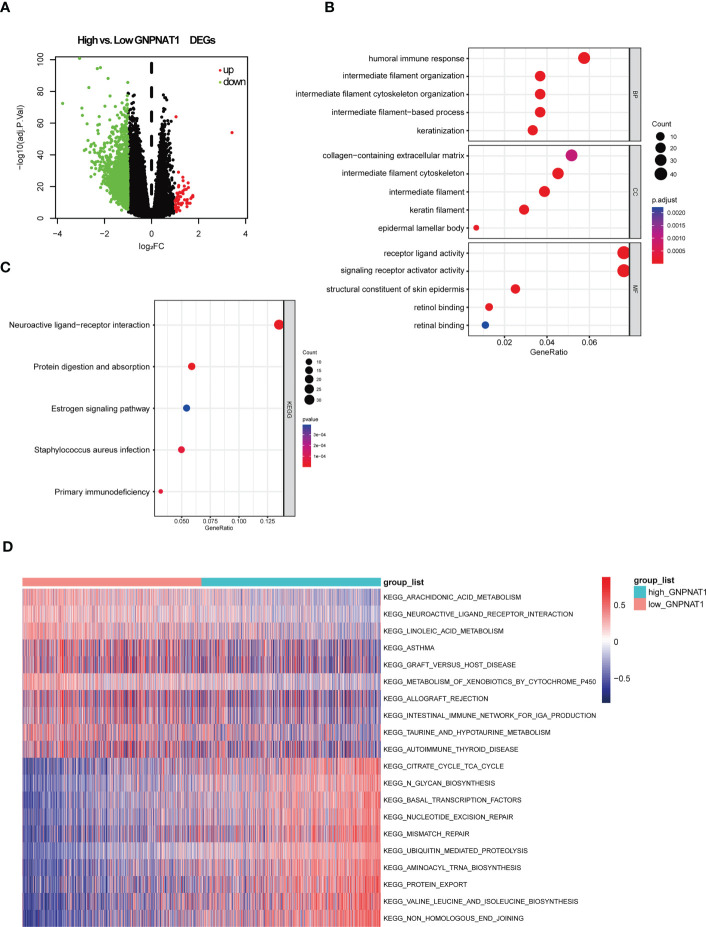
GNPNAT1-related DEGs and functional enrichment analysis of GNPNAT1 in breast cancer using GO, KEGG and GSVA. **(A)** Volcano plot of DEGs in high GNPNAT1 group in TCGA-BRCA cohort compared with low GNPNAT1 group. **(B)** Dot plot of the DEGs involving GO terms. **(C)** Dot plot of the DEGs involving KEGG pathways. **(D)** Heatmap of differentially expressed KEGG pathways through GSVA.

To elucidate the potential mechanism of GNPNAT1 in BRCA patients, we divided 1083 BRCA patients into two groups by the median expression level of GNPNAT1. We totally identified 1877 DEGs including 57 up- and 1820 down- regulation DEGs (**Figure 7A**). Then, DEGs were analyzed using GO terms that provide a context of cellular component (CC), molecular function (MF), and biological process (BP). The results revealed that DEGs were enriched in different GO terms such as humoral immune response, intermediate filament−based process and receptor ligand activity (**Figure 7B**). The KEGG pathway enrichment analysis revealed that neuroactive ligand−receptor interaction, protein digestion and absorption and estrogen signaling pathway is enriched in DEGs (**Figure 7C**).

Additionally, GSVA was conducted to find specific pathways for high GNPNAT1 expression group and low GNPNAT1 expression group. GSVA results showed that pathways of non-homologous end joining (NHEJ), mismatch repair, nucleotide excision repair, citrate cycle, N-Glycan biosynthesis and basal transcription factors were significantly enriched in GNPNAT1 high expression group, while arachidonic acid metabolism, linoleic acid metabolism and intestinal immune network for IgA production were enriched in GNPNAT1 low expression group (**Figure 7D**).

In conclusion, more metabolism-related and DNA repair processes were enriched in the high GNPNAT1 expression group, and more immune-related biological processes were found to be enriched in the low GNPNAT1 expression group, suggesting that the high expression of GNPNAT1 conferred an increased tumor proliferation and a decreased immune phenotype in BRCA.

### 3.6 GNPNAT1 and the immune microenvironment in BRCA

To further investigate the relation between the expression of GNPNAT1 and the decreased immune phenotype, we used ESTIMATE, CIBERSORT algorithm and TISIDB database to assess the immune level in BRCA cohort of TCGA.

**Figure 8:**
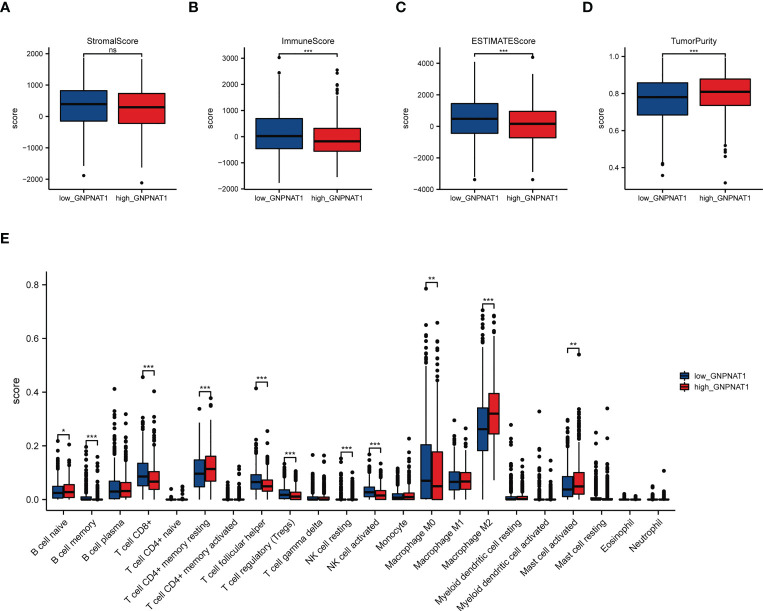
Correlation of GNPNAT1 expression with immune infiltration level in BRCA. **(A)** Stromal scores, **(B)** Immune scores, **(C)** ESTIMATE scores, and **(D)** tumor purity between high GNPNAT1 expression group and low GNPNAT1 expression group (Wilcoxon rank sum test). **(E)** CIBERSORT analysis between the high GNPNAT1 and low GNPNAT1 group (Wilcoxon rank sum test). (*p-value < 0.05; **p-value <0.01; ***p-value <0.001). ns, not significant.

ESTIMATE is a method that uses gene expression signatures to evaluate the levels of immune cell infiltration, stromal content score, stromal-immune comprehensive score and tumor purity for each BRCA sample. The results of the ESTIMATE analyses suggested that the high GNPNAT1 expression group had lower immune score and ESTIMATE score than the low GNPNAT1 expression group, while the tumor purity score was higher (**Figures 8A–D**).

Then, we estimated the proportions of 22 distinct immune cell types using the CIBERSORT algorithm. The CIBRSORT analyses showed that expression of GNPNAT1 has significant positive correlations with T cells CD4 memory resting, macrophages M2 and mast cells resting in BRCA, and has negative correlations with B cells memory, T cells CD8, T cells follicular helper, T cells regulator, NK cells activated (**Figure 8E**).

**Figure 9:**
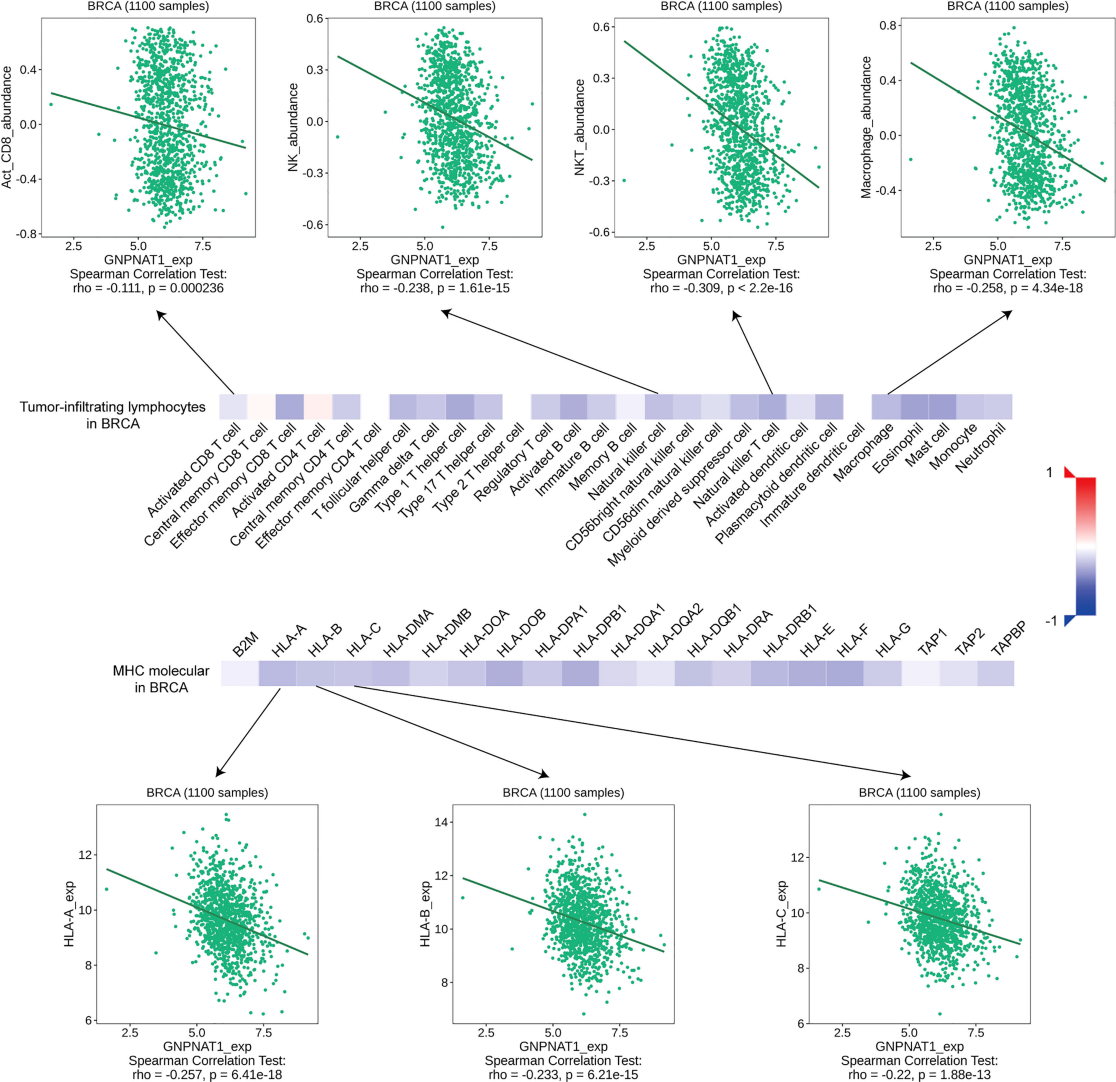
Correlation of GNPNAT1 expression with TILs abundance and MHC molecule expression in BRCA from TISIDB database (Spearman correlation test).

Furthermore, we investigated the relations between abundance of TILs, MHC molecule and expression of GNPNAT1 via TISIDB database. As shown in **Figure 9**, the expression of GNPNAT1 was negatively associated with almost all TILs abundance and MHC molecule expression. Representative images of Pearson correlation analysis were also presented.

**Figure 10:**
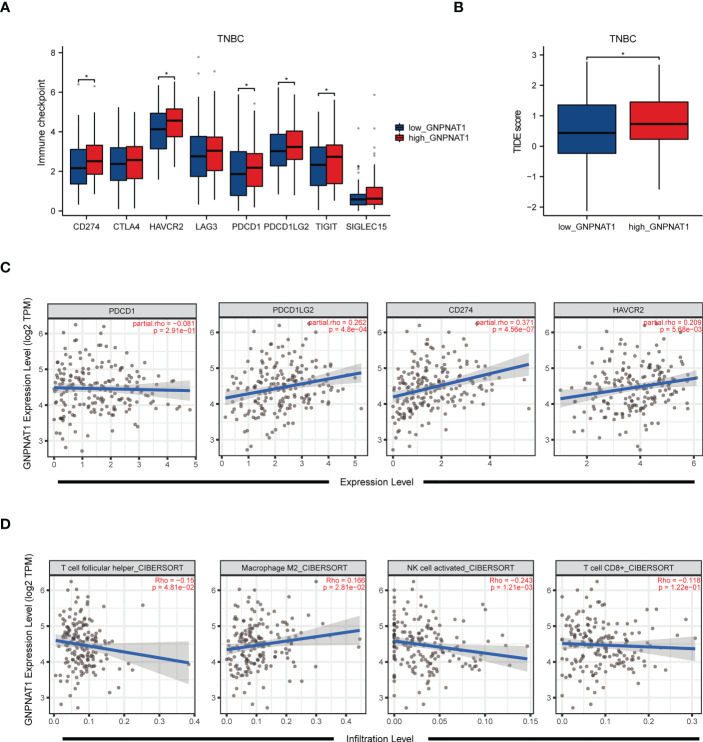
Correlation of GNPNAT1 expression with immunotherapy response and immune microenvironment in TNBC. **(A, B)** Box plot showed the expression of immune checkpoints and TIDE score in patients with different expression of GNPNAT1 in TNBC patients (Wilcoxon rank sum test). **(C, D)** Spearman correlation analysis between expression of GNPNAT1 and expression levels of immune checkpoints and infiltration levels of immune cells (Spearman correlation test). (*p-value < 0.05).

Nowadays immunotherapy targeting immune checkpoint is a rising field of TNBC treatment, so we also want to figure out the correlation between the expression of GNPNAT1 and the efficacy of immune checkpoint inhibitors (ICIs) in TNBC patients. In TNBC from TCGA cohort, the group with high expression of GNPNAT1 had increasing expression level of inhibitory immune checkpoints expression, such as CD274, Hepatitis A Virus Cellular Receptor 2 (HAVCR2), Programmed cell death1 (PDCD1), programmed cell death 1 ligand 2 (PDCD1LG2) and T cell immunoreceptor with Ig and ITIM domains (TIGIT) (**Figure 10A**), and also had a poor response to immunotherapy via TIDE algorithm (**Figure 10B**). Spearman correlation analysis revealed that the expression of GNPNAT1 are positively correlated with the expression of CD274, PDCDLG2 and HAVCR2 (**Figure 10C**), and the expression of GNPNAT1 was negatively correlated with the infiltration of follicular helper T cell, activated NK cell and CD8^+^ T cell in TNBC patients (**Figure 10D**).

Taken together, the expression of GNPNAT1 was negatively associated with immune cell recruitment in BRCA, which possibly altered the composition of immune cells in the tumor environment and thereby affected the progression of BRCA patients. Patients with up-regulated GNPNAT1 had low level of TILs infiltration and MHC abundance, and TNBC patients with up-regulated GNPNAT1 had poor efficacy of immunotherapy in terms of ICIs.

## 4 Discussion

In the previous study, researchers observed that GNPNAT1 was significantly up-regulated in lung adenocarcinoma (9–11), and overexpression of GNPNAT1 led to promote proliferative and metastatic ability of A549, a non-small cell lung cancer cells (22). Additionally, intracellular levels of GNPNAT1 in ER positive breast cancer cell lines had the potential to predict sensitivity to radiotherapy (8). However, there has no study regarding the clinical role and potential mechanism of GNPNAT1 in BRCA.

In this study, we demonstrated that GNPNAT1 was significantly overexpressed in nine out of the 23 human cancer tissues. Based on the ROC analyses, the diagnostic efficacy of GNPNAT1 was proved in different cohorts, and AUC value highlighted the significant differences between the normal tissues and different tumor stages. In addition, GNPNAT1 was found as an independent predictor for overall survival in BRCA patients. The following analyses also confirmed that higher GNPNAT1 expression in BRCA was related to more unfavorable prognosis in BRCA patients. A high GNPNAT1 expression was observed in BRCA from cell lines and clinical samples as well as TCGA and GEO datasets.

GNPNAT1 is a protein with a crucial role in HBP, and HBP is a branch of carbohydrate metabolism which links glucose, amino acid, nucleotide and fatty acid metabolism. Except for increasing glucose consumption, tumor cells also enhanced the demand for glutamine, an essential substrate for HBP (23). Furthermore, the final product of HBP, UDP-GlcNAc, is an essential cell signal regulator and contributes to tumor growth. UDP-GlcNAc is then used for N-linked and O-linked glycosylation and for O-GlcNAcylation modification of nuclear and cytoplasmic proteins (24). O-GlcNAcylation, one type of post-translational modification, was prevalent in tumors, and enhancing O-GlcNAcylation promotes DNA repair, proliferation, epithelial-mesenchymal transition, invasion and metastasis in BRCA (25–27). A study also reported that inhibition of HBP causes breast cancer growth arrest and apoptosis (28). Similar to this, our *in vitro* experiments have confirmed that knocking down GNPNAT1 in breast cancer cell lines results in reduced proliferation and invasion capabilities of breast cancer cells. Similar to these, our *in vitro* experiments have indicated that knocking down GNPNAT1 in breast cancer cell lines results in decreased proliferation and invasion capabilities of breast cancer cells. Our GSVA results also reminded that high GNPNAT1 expression group was mainly enriched in the TCA cycle, NHEJ, mismatch repair, ubiquitin-mediated proteolysis and amino acid biosynthesis, in line with the above study, which proved our functional enrichment results are meaningful.

Tumor microenvironment, the basis of tumor growth and development, was infiltrated by immune cells (29). Our results reveal that GNPNAT1 expression is negatively correlated with almost all TILs infiltration and MHCs abundance, which may suggest that high levels of GNPNAT1 reduce the infiltration and antigen presentation of immune cells in BRCA. Tumor-associated macrophages playing a key role in shaping the tumor microenvironment (30). The prior reports pointed out that glucose metabolism promotes O-GlcNAcylation in macrophages to enhanced tumor invasion (31), and decreasing of O-GlcNAcylation lead to mitigating the immune-suppressive program of M2 macrophages (32). Similarly, we explored that increasing expression of GNPNAT1 has a positive correlation with M2 macrophages, while has a negative correlation with total macrophages, which possibly alters the composition of macrophages in the tumor environment and thereby affected the progression of BRCA patients.

ICIs enhance antitumor immunity mainly through overcome tumor cell-mediated immune inactivation, reverses immune tolerance, restores or strengthens the patient’s own anti-tumor immunity (33). The clinical experiments with the ICIs, such as anti-PD1/PD-L1 and anti-CTLA-4, alone or in combination with chemotherapy, has performed for TNBC patients, and some ICIs were approved for use as a means of clinical therapy (34). Increasing sensitivity of immunotherapy and predicting the outcome of immunotherapy are the hot topics recently. Previous study already proved that targeting HBP sensitizes pancreatic cancer to anti-PD1 therapy in pancreatic cancer (35), and O-GlcNAcylation inhibition activates T cell-mediated antitumor immunity *in vitro (* 36). Our results point out that, in TNBC patients, high GNPNAT1 expression are positively correlated with inhibitory immune checkpoints expression, such as CD274, PDCD1, PDCD1LG2, TIGIT, HAVCR2. Patients with high expression of immune checkpoints are more suitable for immunotherapy, but the results of TIDE algorithm revealed that TNBC patients with high GNPNAT1 expression has poor immunotherapy efficacy. Hence, we speculate that the targeting GNPNAT1 become an effective combined method to sensitize immunotherapy, and it even become a brand-new and universal anti-tumor therapy target in TNBC.

## 5 Conclusion

In summary, GNPNAT1 was high expressed in BRCA, and the up-regulation was related to a poor prognosis. Our study suggests that GNPNAT1 may serve as a diagnostic and prognostic biomarker and novel target for intervention in BRCA patients in the future. Our future study will focus on the mechanism of GNPNAT1 in BRCA.

## Data availability statement

The datasets presented in this study can be found in online repositories. The names of the repository/repositories and accession number(s) can be found within the article/**Supplementary Material**.

## Author contributions

YZ and RY designed this study. RY, YW, HC, RZ and ZH performed bioinformatic analyses. RY and YZ performed the wet experiments and wrote the first draft of the manuscript. CC and TC funded and revised the manuscript. All authors contributed to the article and approved the submitted version.
