# Supplementary material for: GNPNAT1 is a potential biomarker correlated with immune infiltration and immunotherapy outcome in breast cancer
Source: Front Immunol. 2023 May 5;14:1152678. doi: 10.3389/fimmu.2023.1152678 (PMC10195997; doi:10.3389/fimmu.2023.1152678)

Supplementary Material

GNPNAT1 is a Potential Biomarker Correlated with Immune Infiltration and Cancer Stemness in Breast Cancer

**Renjie Yuan^1†^, Yulu Zhang^1†^, Yange Wang^1^, Hongling Chen^1^, Ruiming Zhang^1^, Zhiyuan Hu^2^, Chengsen Chai^1*^, Tingmei Chen^1*^**

*** Correspondence:**

Tingmei Chen, tingmeichen@cqmu.edu.cn
Chengsen Chai, chengsenchai@cqmu.edu.cn

# Supplementary Figures and Tables

## Supplementary Figure 1

**Supplementary Figure 1.** The Kaplan–Meier curves about the correlation between GNPNAT1 expression and overall survive of (A) all patients, (B)HR positive, (C)HER2 positive, and (D)TNBC of in TCGA database (Log-rank test) via GEPIA 2.


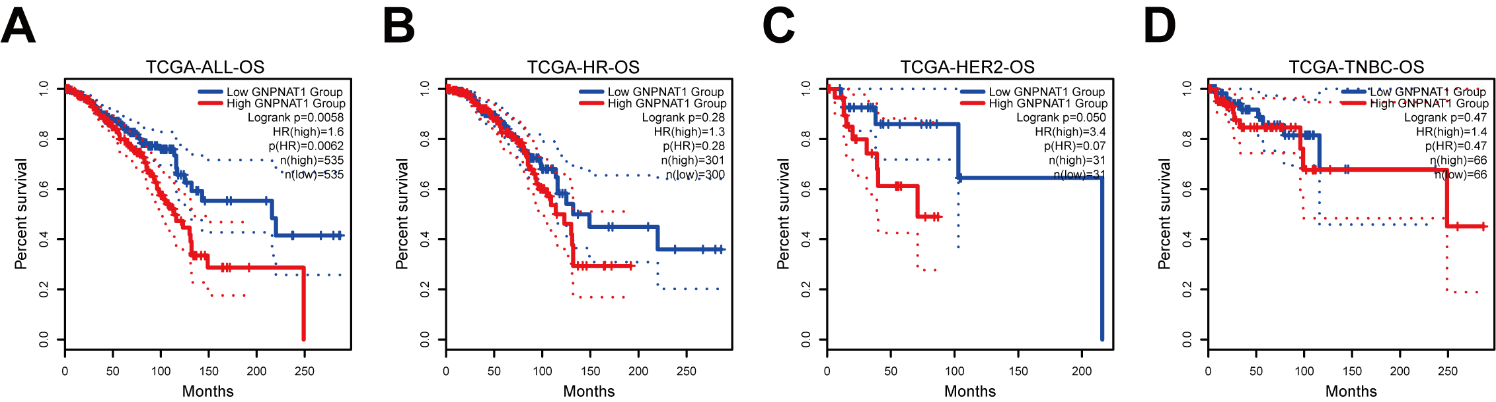

Supplement: Supplementary file 1 [file DataSheet_1.docx]
